# A shared group of bacterial taxa in the duodenal microbiota of undernourished Pakistani children with environmental enteric dysfunction

**DOI:** 10.1128/msphere.00196-24

**Published:** 2024-05-14

**Authors:** Najeeha T. Iqbal, Robert Y. Chen, Nicholas W. Griffin, Matthew C. Hibberd, Aqsa Khalid, Kamran Sadiq, Zehra Jamil, Kumail Ahmed, Junaid Iqbal, Aneeta Hotwani, Furqan Kabir, Najeeb Rahman, Arjumand Rizvi, Romana Idress, Zubair Ahmed, Sheraz Ahmed, Fayaz Umrani, Sana Syed, Sean R. Moore, Asad Ali, Michael J. Barratt, Jeffrey I. Gordon

**Affiliations:** 1 Department of Paediatrics and Child Health, Aga Khan University Hospital, Karachi, Pakistan; 2 Department of Biological and Biomedical Sciences, Aga Khan University Hospital, Karachi, Pakistan; 3 The Edison Family Center for Genome Sciences and Systems Biology, Washington University School of Medicine, St. Louis, Missouri, USA; 4 Center for Gut Microbiome and Nutrition Research, Washington University School of Medicine, St. Louis, Missouri, USA; 5 Department of Pathology and Laboratory Medicine, Aga Khan University Hospital, Karachi, Pakistan; 6 Division of Pediatric Gastroenterology, Hepatology, and Nutrition, Department of Pediatrics, University of Virginia, Charlottesville, Virginia, USA; University of Michigan-Ann Arbor, Ann Arbor, Michigan, USA

**Keywords:** childhood undernutrition, pathogenesis of environmental enteric dysfunction, small intestinal microbiota

## Abstract

Environmental enteric dysfunction (EED) is a subclinical syndrome of altered small intestinal function postulated to be an important contributor to childhood undernutrition. The role of small intestinal bacterial communities in the pathophysiology of EED is poorly defined due to a paucity of studies where there has been a direct collection of small intestinal samples from undernourished children. Sixty-three members of a Pakistani cohort identified as being acutely malnourished between 3 and 6 months of age and whose wasting (weight-for-length *Z*-score [WLZ]) failed to improve after a 2-month nutritional intervention underwent esophagogastroduodenoscopy (EGD). Paired duodenal luminal aspirates and duodenal mucosal biopsies were obtained from 43 children. Duodenal microbiota composition was characterized by sequencing bacterial 16S rRNA gene amplicons. Levels of bacterial taxa (amplicon sequence variants [ASVs]) were referenced to anthropometric indices, histopathologic severity in biopsies, expression of selected genes in the duodenal mucosa, and fecal levels of an immunoinflammatory biomarker (lipocalin-2). A “core” group of eight bacterial ASVs was present in the duodenal samples of 69% of participants. *Streptococcus anginosus* was the most prevalent, followed by *Streptococcus* sp., *Gemella haemolysans*, *Streptococcus australis*, *Granulicatella elegans*, *Granulicatella adiacens,* and *Abiotrophia defectiva*. At the time of EGD, none of the core taxa were significantly correlated with WLZ. Statistically significant correlations were documented between the abundances of *Granulicatella elegans* and *Granulicatella adiacens* and the expression of duodenal mucosal genes involved in immune responses (dual oxidase maturation factor 2, serum amyloid A, and granzyme H). These results suggest that a potential role for members of the oral microbiota in pathogenesis, notably *Streptococcus*, *Gemella,* and *Granulicatella* species, warrants further investigation.

**IMPORTANCE:**

Undernutrition among women and children is a pressing global health problem. Environmental enteric dysfunction (EED) is a disease of the small intestine (SI) associated with impaired gut mucosal barrier function and reduced capacity for nutrient absorption. The cause of EED is ill-defined. One emerging hypothesis is that alterations in the SI microbiota contribute to EED. We performed a culture-independent analysis of the SI microbiota of a cohort of Pakistani children with undernutrition who had failed a standard nutritional intervention, underwent upper gastrointestinal tract endoscopy, and had histologic evidence of EED in their duodenal mucosal biopsies. The results revealed a shared group of bacterial taxa in their duodenums whose absolute abundances were correlated with levels of the expression of genes in the duodenal mucosa that are involved in inflammatory responses. A number of these bacterial taxa are more typically found in the oral microbiota, a finding that has potential physiologic and therapeutic implications.

## INTRODUCTION

Environmental enteric dysfunction (EED), a disorder associated with loss of small intestinal absorptive surface area and mucosal barrier disruption, is postulated to be a contributing factor to childhood undernutrition. The challenge in testing this hypothesis is the difficulty in directly sampling the small intestinal mucosa, as well as a paucity of biomarkers that have been shown to be associated with EED in multiple populations (1).

The role of the small intestinal microbiota in the pathogenesis of EED and stunting was examined in a recent study involving a cohort of 110 Bangladeshi children aged 18 ± 2 months with linear growth faltering who had failed a nutritional intervention and subsequently underwent esophagogastroduodenoscopy (EGD) (2). Histopathologic analysis of their duodenal biopsies indicated that 95% had EED. Matched sets of duodenal mucosal biopsies, duodenal luminal aspirates, and plasma samples were obtained from 38 of these children, only two of whom had normal duodenal mucosal histology. Analysis of bacterial 16S rRNA genes represented in their duodenal aspirates revealed a group of 14 bacterial taxa (16S rRNA amplicon sequence variants, or ASVs) that were shared by >80% of the children. The absolute abundances of organisms in this group, which are not typically categorized as enteropathogens, were significantly negatively correlated with linear growth (length-for-age *Z* score [LAZ]) and significantly positively correlated with the levels of duodenal and plasma protein biomarkers and mediators of immunoinflammatory responses (2). Moreover, colonization of germ-free mice with a consortium of bacteria cultured from these duodenal aspirates, including strains representing 9 of the 14 core group members, produced a small intestinal enteropathy in recipient animals fed with a diet resembling that consumed by children in the study cohort (2). These findings provided evidence that there may be a causal relationship between components of the small intestinal microbiota, enteropathy, and stunting.

Here, we examine a cohort of Pakistani children with acute malnutrition (wasting as defined by weight-for-length *Z* score [WLZ]) who had undergone EGD after they failed a nutritional intervention (3). This report focuses on children with biopsy-confirmed EED; specifically, characterization of the bacterial composition of their duodenal microbiota and the extent to which the absolute abundances of their duodenal bacterial community members correlate with a variety of host parameters.

## MATERIALS AND METHODS

### Study design and biospecimen collection

The SEEM study was conducted between March 2016 and March 2019 in Matiari, a rural site in Pakistan. SEEM was a prospective longitudinal cohort study of children followed from birth through 24 months of age. The study design has been described previously (3). A total of 365 malnourished children, defined as having a WLZ<−2.0, and between ages 3 and 6 months, were enrolled. At enrollment, parents/guardians received educational counseling focusing on breastfeeding and complementary feeding practices. Anthropometric measurements were taken at enrollment and then monthly thereafter (length at 1 mm precision using a rigid length board with movable footpiece; weight at 20 g precision using a TANITA 1584 electronic scale). A nutritional intervention was carried out if WLZ was <−2.0 at 9 months of age; the protocol involved community management with the provision of Acha Mum, a local chickpea-based ready-to-use supplementary food, and weekly monitoring of compliance. Children with WLZ between −2 and −3 received one sachet of Acha Mum per day for 2 months (4). Those who (i) failed to respond to the nutritional intervention (defined as having a WLZ <−2.0), (ii) lacked evidence of celiac disease (based on the measurement of anti-tTg-IgA), and (iii) had complete blood counts, total serum IgA, serum electrolyte, and creatinine values that ruled out other causes of growth faltering were deemed eligible for EGD. Consent for EGD was obtained from the parents/guardians of 63 of these children. Blood samples were collected before endoscopy. Mucosal biopsies from the D2 region of the duodenum were collected at the time of EGD from each of the 63 children enrolled; duodenal aspirates were collected from 43 of these children, while 20 had insufficient duodenal fluid to be recovered. All specimens were stored at −80°C. Biopsies were used for histopathological grading of EED, while the bacterial content and enteropathogen composition of aspirates were assessed by sequencing amplicons of the V4 16S rRNA gene and with TaqMan Array Cards (TAC), respectively (see below).

### Histopathologic grading of EED

Histopathology in duodenal biopsies was assessed using scoring criteria developed by the EED Biopsy Initiative (5). This scoring system is based on parameters that include villous architecture, goblet cell depletion, Paneth cell depletion, number of intraepithelial lymphocytes (IELs), and number of intramucosal Brunner glands.

### Bacterial 16S rRNA amplicon sequencing and analyses of ASVs

Duodenal aspirates obtained from children were used to generate PCR amplicons from variable region 4 of the bacterial 16S ribosomal RNA gene according to a previously described protocol (2). Briefly, 50 µL of each duodenal aspirate was aliquoted into a skirted PCR plate (MultiMax 2668). A total of 1.1 × 10^6^ *Alicyclobacillus acidiphilus* cells were added to each well as a spike-in control to allow for the quantification of the absolute abundances of community members. The resulting mixture was incubated in proteinase K (1 µg/µL; Thermo Fisher Scientific) for 10 h at 65°C, followed by a 10-min incubation at 95°C to inactivate the enzyme. DNA was isolated by phenol-chloroform extraction, V4-16S rDNA amplicons were generated, amplicon libraries were produced as previously described (2), and the libraries were sequenced (Illumina MiSeq instrument; 2 × 250 paired-end reads; 4.8 ± 1.2 × 10^5^ [mean ± SD] reads/sample).

Read pairs were trimmed to remove low-quality 3′ bases and filtered to remove unpaired reads using bbtools (v37.02). Pre-processed reads were analyzed in R (v4.2.2) using the Divisive Amplicon Denoising Algorithm (DADA2) to create abundance profiles for ASVs in each sample. Taxonomic assignments were performed for each ASV using the “assignTaxonomy” function in DADA2 and the SILVA database (build 138) (6). The QIIME2 “q2-phylogeny” plugin was used to generate a phylogenetic tree based on the ASV sequences (7, 8). Alpha diversity metrics (Shannon and Simpsons) were defined using the plot richness function in Phyloseq. All data objects (ASV abundances, taxonomic assignments, the phylogenetic tree, and sample metadata) were combined into a Phyloseq object for downstream analysis. For statistical analyses, absolute abundances were normalized using DESeq2 to adjust for library size and then log_10_-transformed (2).

The correspondence of ASV sequences between the current and prior studies of duodenal aspirates was determined by matching identical sequences in each data set. ASVs identified in the current study and ASVs identified in the prior Bangladesh Environmental Enteric Dysfunction (BEED) study (2) were generated and processed identically; therefore, correspondence was determined by searching the SEEM data set for identical sequences in the BEED data set. To determine correspondence between SEEM aspirate ASVs and sequences in oral taxon reference databases, full-length 16S rRNA data sets were downloaded from the CORE oral microbiome database (http://microbiome.osu.edu; “CORE.fna.gz,” *n* = 1,262 sequences) and the expanded human oral microbiome database (eHOMD; https://www.homd.org/download; “HOMD_16S_rRNA_RefSeq_V15.23.p9.fasta,” *n* = 1,015 sequences) (9, 10). A blast-compatible database was created for each reference data set, and the V4 16S rRNA ASVs from the SEEM study were searched against each database using blastn (11). Results in tabular form were processed in R (v4.3.2) to remove alignments that were not full length or that contained mismatches.

### Enteropathogen assays

A previously described TAC assay (12) and the QuantStudio 7 Flex platform were used to survey duodenal aspirate samples for the presence/abundance of 44 enteropathogens. The following thresholds were set to score enteropathogens as “present” in a given sample: (i) Ct value less than 35.0, (ii) negative reference extraction blank for each target/sample, and (iii) Ct value for internal controls (MS2 and PhHV) less than 35.0.

### Fecal protein assays

Fecal samples collected within 2.12 ± 0.89 days (mean ± SD) either pre- or post-EGD were used to measure lipocalin-2 (LCN2) levels. Assays were performed using a commercially available enzyme-linked immunosorbent assay (Duoset ELISA cat # DY1757; R&D, Minneapolis, MN, USA).

### Statistical analysis

The strength of the associations between the absolute abundances of members of the core group of duodenal ASVs, anthropometric indices, histopathologic scores for mucosal biopsies, duodenal enteropathogens, fecal lipocalin-2 measurement, and expression of 13 EED-associated genes in duodenal biopsies were determined using the Pearson correlation coefficient. The significance of the relationship between levels of bacterial enteropathogens in aspirates and fecal lipocalin-2 biomarker collected at the time of EGD was assessed using Pearson rank correlation. All statistical analyses were performed using RStudio R (v4.2.2). A *P*-value < 0.05 was considered statistically significant.

## RESULTS

### Study population

A prospective, longitudinal study of 416 children who lived in a rural site in Pakistan (Matiari) and were followed by monthly anthropometry from birth identified 365 participants who manifested wasting between 3 and 6 months of age. A subset of 63 children were selected for EGD. These children had not responded to an initial nutritional intervention involving a locally produced ready-to-use supplementary food (RUSF), Acha Mum 4) (Table S1). This RUSF was first administered for 57.8 ± 6.3 days at 9.89 ± 0.99 months of age (mean ± SD), because their WLZ scores were below −2.0. The intervention had to be repeated in 12% of these children owing to the fact that they failed to respond to the initial intervention. Five children who received this second round of intervention also underwent EGD; the interval between their first and second intervention ranged from 4 to 8 months. Table S2A and B summarize the clinical characteristics of the participants who underwent EGD plus the timing of RUSF treatments and the anthropometric responses of each child. Matched duodenal biopsies and duodenal aspirates were obtained from 43 of the 63 children (insufficient duodenal fluid for analysis was collected from the other 20 children). Their age at the time of EGD was 19 ± 3.8 months (mean ± SD) and 35% were female (Table 1).

**TABLE 1:** Clinical characteristics of children who failed nutritional intervention and underwent EGD

Demographic features	Participants (*n* = 43)
Age in months at the time of EGD (mean ± SD)	19 ± 3.8
Number of females (%)	15 (35)
Response to nutritional intervention (NI)- (beginning to end of last day of supplementation)	Δ (mean at the beginning, mean at the last day of NI)
ΔWLZ	0.20 (−2.64,−2.44)
ΔWAZ	0.14 (−3.49,−3.35)
ΔLAZ	−0.19 (−2.52,−2.71)
Anthropometry at EGD	Mean ± SD
WLZ at endoscopy	−2.17 ± 0.70
WAZ at endoscopy	−3.03 ± 0.78
LAZ at endoscopy	−2.88 ± 1.16
Duodenal biopsy histopathology	
Histology score of biopsy (0–12) (mean ± SD)	5.86 ± 2.5
Participants with no evidence of EED (%)	0 (0)
Participants with mild EED [score=0–5] (%)	20 (46.5)
Participants with moderate EED [score=6] (%)	9 (21)
Participants with severe EED [>6] (%)	14 (32.5)
Fecal biomarkers at EGD	Mean (min, max)
Lipocalin-2 (ng/mL)	3,247 (11, 19,243)

### Histopathologic assessment

The histopathological severity of EED in the duodenal mucosal biopsies was defined based on several parameters including measurements of villous blunting, IELs, goblet and Paneth cell depletion, and intramucosal Brunner’s glands (5). There was no significant correlation between WLZ at the time of EGD and the histopathological score (Spearman *r* = 0.057; *P* = 0.72). There were also no significant correlations between any of the individual parameters of the scoring scale and WLZ at endoscopy (see Table S2A).

### Bacterial content of duodenal aspirates

**Fig 1:**
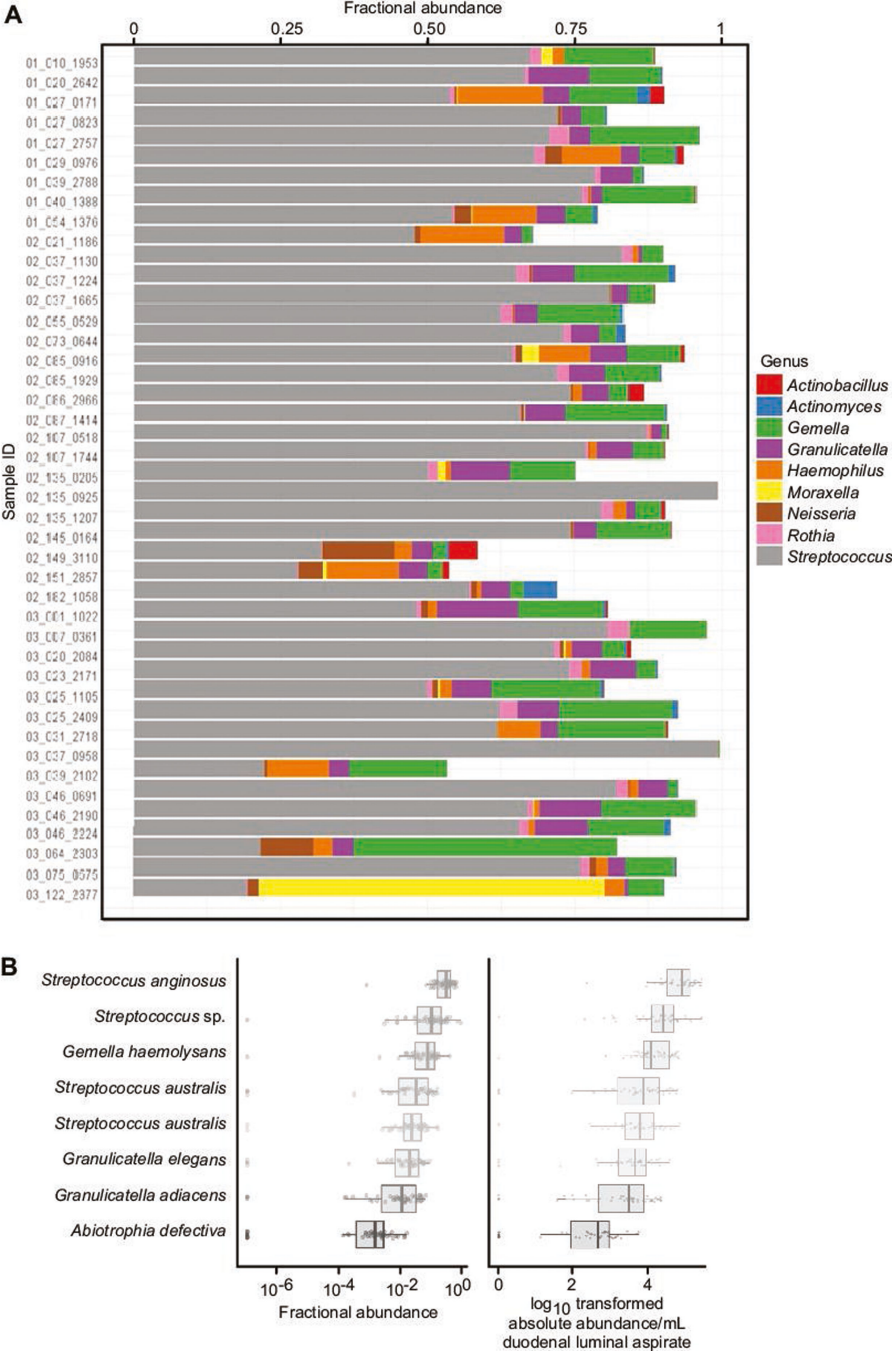
Defining a core group of bacterial taxa (ASVs) in duodenal aspirates. (**A**) Genus-level assignments and fractional abundances of bacterial taxa present in duodenal aspirates obtained from each of the 43 children with EED. Bacteria classified as *Streptococcus* (gray bars)*, Gamella* (green), and *Granulicatella* (purple) are the most consistently observed and abundant across samples. (**B**) Boxplots of the relative and absolute abundances of the eight “core” taxa (ASVs) identified in the 43 duodenal aspirates.

Bacterial taxa present in duodenal aspirates were identified as V4-16S rRNA ASVs. A total of 278 ASVs satisfied our threshold criteria of being present at a relative abundance of ≥0.01% in at least one aspirate sample (Table S3A through D). Alpha diversity metrics did not correlate with histology scores (Shannon *r* = −0.107, *P* = 0.49; Simpsons *r* = −0.106, *P* = 0.49 [Pearson correlation coefficient]) (Table S3E). The genera with the highest fractional abundances across the 43 aspirate samples are shown in Fig. 1A. Eight “core” taxa were identified based on having a relative abundance of ≥0.01% in at least 80% of aspirates. The most prevalent taxa were ASV1 (*Streptococcus anginosus*, present in 100% of aspirates), followed by ASV2 (*Streptococcus* sp., 97%), ASV5 (*Gemella haemolysans,* 97%), ASV6 (*Streptococcus australis* sp. *infantis*, 91%), ASV7 (*Streptococcus australis* sp. *mitis,* 95%), ASV9 (*Granulicatella elegans*, 91%), ASV10 (*Granulicatella adiacens*, 93%), and ASV32 (*Abiotrophia defectiva*, 81%) (see Fig. 1B; Table S3C and D for their absolute and relative abundances). Of the 43 aspirates, 30 (69%) had all eight core taxa. The absolute abundance of the group of eight core taxa was not correlated with WLZ at the time of EGD (*r* = 0.2, *P* = 0.4). Phylogenetic comparisons of the 278 ASVs identified in the SEEM and 164 ASVs similarly identified in the Bangladeshi Environmental Enteric Dysfunction study (2) revealed that 42 were shared (i.e., their ASV sequences were identical) (Table S3F). This set of shared taxa included three of the eight “core” ASVs from the current study, namely, a *Streptococcus* sp., *Granulicatella elegans,* and a *Gemella* sp. Moreover, 13 of the 14 BEED core ASVs were present in the set of SEEM ASVs (Table S3F).

**Fig 2:**
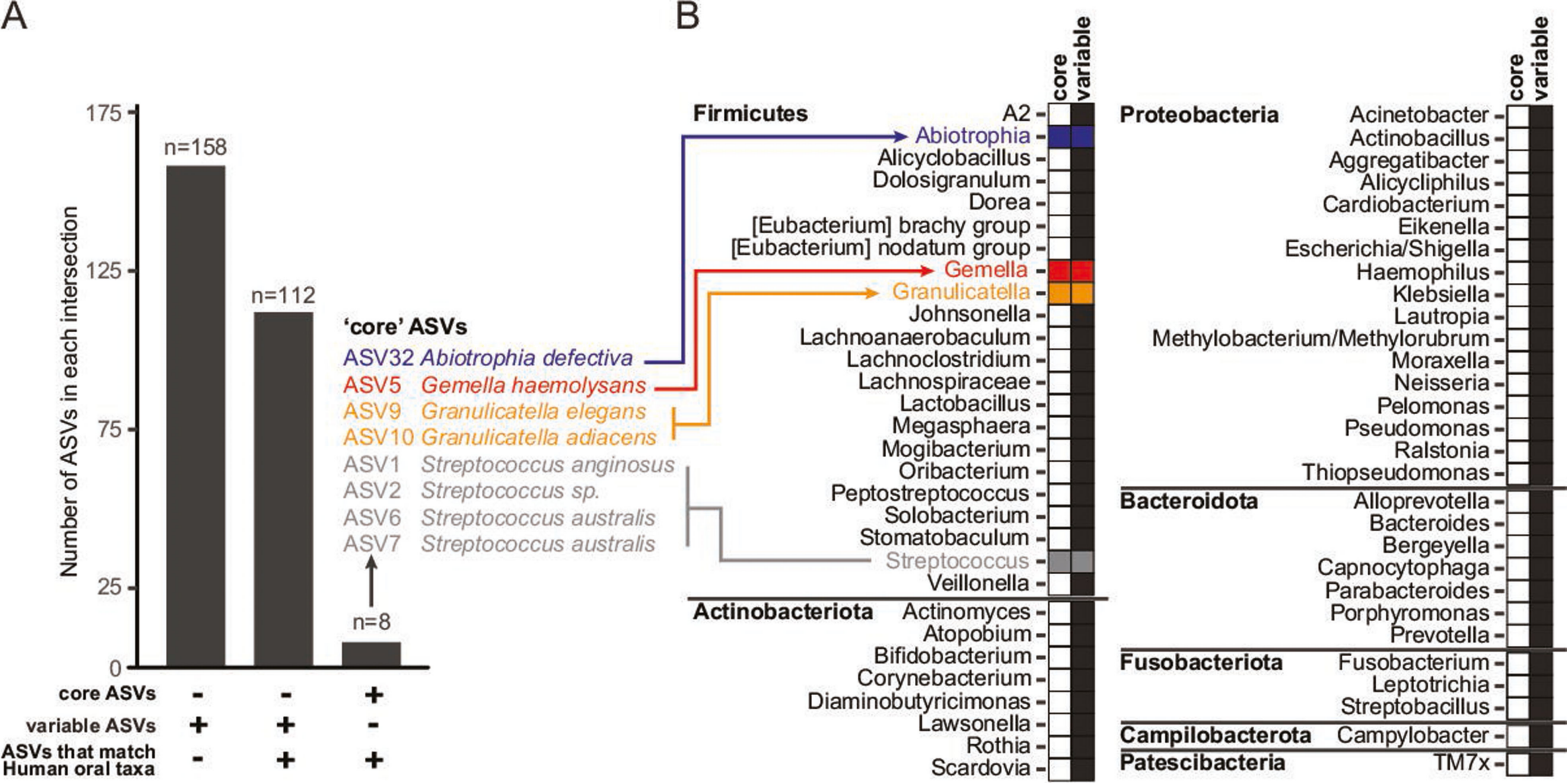
Correspondence between duodenal aspirate ASVs and members of the oral microbiota. Assignment of duodenal aspirate ASVs as candidate oral taxa was determined by aligning ASVs to two reference databases of oral taxa (see Materials and Methods). (**A**) The number of ASVs assigned to “core” or “variable” (abundance) taxa that also aligned with an oral taxon sequence in one or both reference databases. (**B**) Genus assignments of ASVs from the “core” or “variable” ASV sets that match sequences from oral taxa. Filled boxes indicate whether ASVs assigned to a given genus were part of the core set (blue, red, orange, or gray boxes), the set with variable representation (black boxes), or both.

We next sought to determine the associations between ASVs identified in duodenal aspirate sequencing efforts and taxa commonly found in the oral versus duodenal environment. To do so, we cross-referenced our duodenal aspirate ASV sequences for correspondence to full-length 16S rRNA reference sequences downloaded from either the CORE human oral microbiome database (9) or the expanded human oral microbiome database (10) (see Materials and Methods). As depicted in Fig. 2, 120 of 278 (43%) ASVs were aligned to one or more reference sequences belonging to one or both of these reference databases, whereas 158 (57%) were not (Table S3G). Interestingly, all eight “core” ASVs identified from duodenal aspirates in the current study were annotated as oral taxa using both databases, a number significantly greater than expected by chance alone (*P* = 0.0011, Fisher’s exact test).

**Fig 3:**
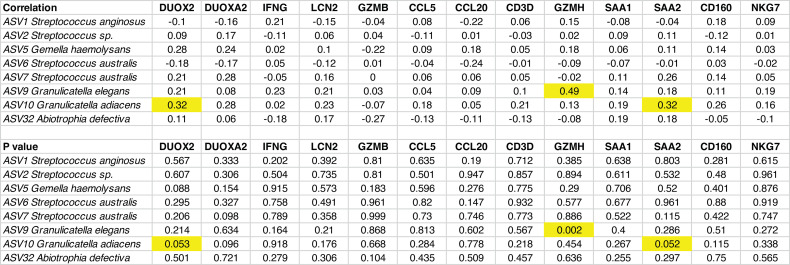
Correlations between absolute abundances of duodenal core taxa and duodenal expression of EED-associated genes. Shown are Pearson correlation coefficients between the absolute abundance of a core taxon (ASV) and duodenal mucosal expression of an EED-associated gene. Correlations that were statistically significant (*P*-value < 0.05) are highlighted in yellow. Dual oxidase 2 (DUOX2), dual oxidase maturation factor 2 (DUOXA2), interferon-gamma (IFNG), lipocalin-2 (LCN2), granzyme B (GZMB), C-C motif chemokine ligand 5 (CCL5), C-C motif chemokine ligand 20 (CCL20), CD3 delta subunit of T-cell receptor complex (CD3D), granzyme H (GZMH), serum amyloid A1 (SAA1), serum amyloid A2 (SAA2), CD160 (CD160), and natural killer cell granule protein 7 (NKG7).

Duodenal aspirates were also tested for the presence of 44 enteropathogens using a TAC qPCR assay (12, 13). This assay has been used to quantify pathogenic bacteria, viruses, protists, and helminths present in fecal and duodenal samples in several studies in low- and middle-income countries (12, 13). Across the cohort, we detected a total of 13 distinct enteropathogens (Table S4), with by far the most common being *Giardia*, which was present in 29 of the 43 aspirates. The number of unique pathogens in each duodenal sample was 1.49 ± 1.33 (mean ± SD), though the number of pathogens detected was not correlated with the histology score (*r* = 0.111, *P* = 0.48). There was also no significant correlation between levels of any one of the 44 enteropathogens and fecal levels of lipocalin-2 an immunoinflammatory biomarker associated with EED (14) that was quantified by ELISA in fecal samples collected 2–5 days prior to endoscopy (Table S5). In addition, there were no statistically significant correlations between fecal levels of lipocalin-2 and any of the core taxa, either individually or as a group. Nonetheless, correlations between the absolute abundances of members of the core taxa and gut inflammation were observed when we considered levels of expression of 13 genes in an adjacent duodenal biopsy. These genes were previously identified as being expressed at significantly higher levels in biopsies adjacent to the one selected here for histopathologic analysis compared to their expression in duodenal biopsies obtained from 2-year-old USA children who had undergone EGD for Celiac disease and had no histopathologic evidence of EED (15). The absolute abundance of *Granulicatella adiacens* was significantly positively correlated with levels of expression of (i) dual oxidase type 2 (DUOX2; Pearson *r* = 0.32; *P* = 0.05), an enzyme whose increased expression is associated with epithelial barrier dysfunction and production of cytokines/chemokines, and (ii) serum amyloid A2 (SAA2; *r =* 0.32; *P* = 0.05), an acute phase protein. In addition, the absolute abundance of *Granulicatella elegans* was significantly positively correlated with the expression of the serine protease granzyme H (GZMH; *r =* 0.49; *P* = 0.002) (Fig. 3).

## DISCUSSION

We have defined a group of eight bacterial taxa (ASVs) that were shared across the duodenal microbiota of >80% of members of a cohort of 43 undernourished Pakistani children with EED. *Streptococcus anginosus* was the most prevalent and was represented in all 43 duodenal aspirate samples. This taxon is a member of the *Streptococcus anginosus* group, which comprises *S. anginosus, Streptococcus constellatus*, and *Streptococcus intermedius* (16, 17), all of which are typically found in the oropharyngeal microbiota. We did not observe a relationship between the absolute abundances of these core taxa and WLZ scores at the time of EGD in our cohort of undernourished children. However, in this regard, our study was limited for ethical reasons since we were not able to perform EGD and analyze the duodenal microbiota of healthy children (with normal anthropometry). Nevertheless, our analyses identified statistically significant positive correlations between the absolute abundances of two core taxa, *Granulicatella adiacens* (18) and *Abiotrophia defectiva* (19), with an established biomarker of EED (lipocalin-2) in pre-endoscopy fecal samples. A strong positive correlation was also observed between the abundances of *Granulicatella adiacens* and *Granulicatella elegans* and the expression of genes in the duodenal mucosa associated with immune activation/inflammation, DUOX2, SAA2, and GZMH (15). This association between the duodenal microbiota and inflammation was also documented in the Bangladesh Environmental Enteric Dysfunction (BEED) study, where the absolute abundance of an ASV assigned to *Granulicatella elegans* was strongly correlated with duodenal mucosal levels of chitinase-3-like protein 1 (CHI3L1), a secreted glycoprotein biomarker of inflammation, as well as lipocalin-2 (2). “Nutritionally variant streptococci” include *Granulicatella adiacens*, *Granulicatella elegans, Gemella haemolysans,* and *Abiotrophia defectiva*; they are also members of the oral microbiota (20).

These findings support a view of EED as representing, at least in part, decompartmentalization of the gut microbiota with components of the oral microbiota establishing themselves within the small intestine (21). As such, our study underscores the importance of characterizing the oral microbiota in children with EED, their mothers, and their healthy counterparts living in areas where the burden of undernutrition is great. Establishing a causal link between the organisms identified in this study and the pathogenesis of EED could involve the development of collections of cultured genome-sequenced bacteria from duodenal aspirates as well as the oral microbiota of children with EED and their introduction into gnotobiotic mice. This type of approach was previously employed using a collection of 39 bacterial strains cultured from duodenal aspirates obtained during EGD from stunted children in the BEED study with histopathological evidence of EED (2). Gnotobiotic mice that received this bacterial consortium developed an enteropathy with features similar to those in the Bangladeshi children whose SI microbiota were used for transplantation (2, 22). Notably, the comparison of the 278 ASVs identified in aspirates from children with EED in the current (SEEM) study and the 164 ASVs reported in the BEED study (2) revealed that 42 were identical, including three of the eight “core” bacteria identified in our study. However, formal confirmation of the ability of the SEEM duodenal microbiota to transmit an enteropathy requires similar preclinical testing to that performed with the strains cultured from the BEED aspirates.

In addition to identifying members of the SEEM SI microbiota that has the capacity to confer enteropathy, tests of causality in these preclinical models offer an opportunity to identify therapeutic targets, including those typically represented in the oral microbiome, and to develop new approaches for treatment as well as diagnosis and prevention.

## Data Availability

V4-16S rRNA amplicon sequences have been deposited in the European Nucleotide Archive under accession number PRJEB75181.

## SUPPLEMENTAL MATERIAL

Supplemental Tables msphere.00196-24-s0001.xlsx Tables S1-S5.

ASM does not own the copyrights to Supplemental Material that may be linked to, or accessed through, an article. The authors have granted ASM a non-exclusive, world-wide license to publish the Supplemental Material files. Please contact the corresponding author directly for reuse.
